# High‐density lipoprotein cholesterol levels are associated with major adverse cardiovascular events in male but not female patients with hypertension

**DOI:** 10.1002/clc.23606

**Published:** 2021-03-30

**Authors:** Xiaopu Wang, Junyu Pei, Keyang Zheng, Xinqun Hu

**Affiliations:** ^1^ Department of Cardiovascular Medicine, The Second Xiangya Hospital Central South University Changsha Hunan China; ^2^ Department of Cardiovascular Medicine, Beijing Anzhen Hospital Capital Medical University Beijing China

**Keywords:** high‐density lipoprotein cholesterol, hypertension, major adverse cardiovascular events, sex difference

## Abstract

**Background:**

The relationship between high‐density lipoprotein cholesterol (HDL‐C) levels and major adverse cardiovascular events (MACEs) in hypertensive patients of different sexes is unclear.

**Hypothesis:**

Sex differences in the relationship between HDL‐C levels and the risk of MACEs among hypertensive patients.

**Methods:**

We performed a post‐hoc analysis of data obtained from the Systolic Blood Pressure Intervention Trial (SPRINT) and explored sex‐based differences in the relationship between HDL‐C levels and MACEs among hypertensive patients using Cox proportional hazards regression.

**Results:**

A total of 9323 hypertensive patients (6016 [64.53%] men and 3307 [35.47%] women) were assessed using SPRINT data. MACEs occurred in 395 (6.57%) men and 166 (5.02%) women after a mean follow‐up of 3.26 years. When HDL‐C levels were used as a continuous covariate, each 10 mg/dl increase in HDL‐C levels decreased the risk of MACEs in men (hazard ratio [HR], 0.78; 95% confidence interval [CI], 0.70–0.88; *p* < .0001). However, HDL‐C levels were not associated with MACEs in female hypertensive patients (HR, 1.02; 95% CI, 0.89–1.16; *p* = .7869). Compared with those in the first quartile, MACEs in the fourth quartile had the lowest risk among male patients (HR, 0.58; 95% CI, 0.41–0.82; *p* = .0023). Female patients in the fourth quartile of HDL‐C levels had an HR of 1.09 for MACEs (95% CI, 0.62–1.93; *p* = .7678). HDL‐C levels were not associated with the risk of MACEs among females.

**Conclusion:**

Among elderly hypertensive patients, higher HDL‐C levels were associated with a lower MACE incidence in men but not in women. Unique identifier: NCT01206062.

## 1 INTRODUCTION

Dyslipidemia is an important risk factor for the development and progression of cardiovascular diseases (CVD). Increased levels of low‐density lipoprotein cholesterol (LDL‐C) and non‐high‐density lipoprotein cholesterol have been reported to be significantly correlated with an increased risk of cardiovascular mortality.^1^, ^2^, ^3^ Nevertheless, even after intensive statin therapy, patients whose LDL‐C levels have reached the target are still at risk for persistent atherosclerosis and cardiovascular events.^1^

In recent years, more attention has been paid to the role of high‐density lipoprotein cholesterol (HDL‐C), and several studies have shown that HDL‐C levels are inversely associated with the risk of cardiovascular events.^4^, ^5^, ^6^ Saito et al. reported that HDL‐C levels are inversely associated with the risk of ischemic stroke.^7^, ^8^ However, recent studies have revealed that high‐density lipoprotein (HDL) may lose its protective effects, leading to atherosclerosis or adverse outcomes.^9^, ^10^, ^11^ In some cases, high HDL‐C levels do not show protection in postmenopausal or older women.^12^, ^13^, ^14^

The relationship between HDL‐C levels and adverse cardiovascular events in hypertensive patients of different sexes is unclear. Hence, we used data from the Systolic Blood Pressure Intervention Trial (SPRINT) to assess sex differences in the relationship between HDL‐C levels and the risk of adverse cardiovascular events among hypertensive patients.

## 2 MATERIALS AND METHODS

We performed a secondary analysis of SPRINT data. We obtained the limited dataset from the National Institutes of Health Biologic Specimen and Data Repository Information Coordinating Center.

### 2.1 Study population

The protocol and main results of the SPRINT have been published.^15^ Briefly, 9361 patients aged 50 years and older with a screening systolic blood pressure (SBP) of 130–180 mmHg were randomized in the SPRINT, which was a two‐arm, multicenter, open‐label, controlled trial. The recruited participants were between 50 and 75 years of age and had at least one of the following: presence of clinical or subclinical CVD other than stroke; chronic kidney disease (CKD; defined as estimated glomerular filtration rate of 20–59 ml/min/1.73 m^2^); Framingham risk score for 10‐year CVD risk ≥15% based on laboratory work conducted in the last 12 months; or patient age of 75 years or older. Eligible participants for the trial were randomized into one of two goals: SBP <120 mmHg for the more intensive goal (intensive arm) and SBP <140 mmHg for the less intensive goal (standard arm). The SPRINT showed that compared with standard management, intensive blood pressure management significantly reduced cardiovascular mortality and all‐cause mortality.

### 2.2 Exposure variables

This study was a post‐hoc analysis of SPRINT data. Data on HDL‐C and LDL‐C levels were collected at baseline. We excluded 38 patients whose data on HDL‐C levels were not available. HDL‐C levels were separated as sex‐specific quartiles because of the nonlinear relationship between HDL‐C levels and adverse outcomes. The primary endpoint of our study was major adverse cardiovascular events (MACEs), defined as a composite of myocardial infarction, stroke, heart failure, and/or death from cardiovascular causes. The definitions of MI, stroke, heart failure, and outcomes were the same as those in the SPRINT and presented elsewhere. The outcomes were adjudicated.

### 2.3 Statistical analysis

The baseline characteristics and outcomes of patients were expressed as frequencies and percentages for categorical variables. Means and *SD*s or median and interquartile ranges were used for continuous variables, depending on whether datasets were normally distributed (assessed using normal Q–Q plots). We used chi‐square analysis to compare categorical variables. We used analysis of variance or the Mann–Whitney U test to compare continuous variables in accordance with the distribution type.

The adjusted variables in this study were selected based on their clinical importance, statistical significance in the univariable analysis, and potential confounding, indicated by estimates that individually changed by at least 10%. We evaluated the relationship between HDL‐C and LDL‐C levels and MACEs using HDL‐C and LDL‐C levels as either continuous or categorical variables. A total of three models were used: (1) model 1, unadjusted; (2) Model 2, adjusted for age, treatment arm, and ethnicity; and (3) Model 3, fully adjusted for age, treatment arm, ethnicity, baseline SBP and diastolic blood pressure, baseline body mass index (BMI), smoking status, CKD subgroup, CVD subgroup, baseline total cholesterol, baseline triglycerides, baseline urine albumin/creatinine ratio, number of antihypertensive agents, aspirin use, and statin use. Using HDL‐C and LDL‐C levels as categorical variables, we used a Cox proportional hazards regression model to calculate the hazard ratios (HR) among categories. The first quartile was used as the reference. We subsequently treated the BMI categories as continuous variables to test the linear trend.

To account for BMI as a continuous variable, we constructed a Cox proportional hazards regression model adjusted for Model 3, in which HDL‐C and LDL‐C levels were used to calculate the HR for outcomes. We further used restricted cubic splines with four knots at the 5th, 35th, 65th, and 95th percentiles to flexibly model the association of HDL‐C and LDL‐C levels with the logarithm of the relative risk of outcomes, in which the mean BMI value served as the reference. Subsequently, a two‐stage linear regression model was used to calculate the threshold effect of the relationship between HDL‐C and LDL‐C levels and MACEs. We used a trial method to determine the threshold value by moving the trial turning point along a predefined interval and selecting the value that provided the maximum model likelihood. Next, we performed log‐likelihood ratio analysis comparing the one‐line linear regression model with the two‐piecewise linear model. We performed interaction and stratified analyses according to treatment arm, age (<75 years and ≥ 75 years), SBP tertile (≤132 mmHg, 132–145 mmHg, and ≥ 145 mmHg), Framingham 10‐year CVD risk score (≤15% and > 15%), smoking status, CVD subgroup, CKD subgroup, black race, aspirin use, and statin use.

All analyses were performed using statistical software packages R (The R Foundation; http://www.R-project.org) and EmpowerStats (X&Y Solutions, Inc., Boston, Massachusetts, USA; http://www.empowerstats.com). *p* values <.05 (two‐sided) were considered statistically significant.

## 3 RESULTS

### 3.1 Baseline characteristics of the included hypertensive patients

Of a total of 9323 hypertensive patients from the SPRINT, 6016 (64.53%) were men and 3307 (35.47%) were women, with a median follow‐up of 3.26 years. After follow‐up, MACEs occurred in 395 (6.57%) men and 166 (5.02%) women. Female patients had higher HDL‐C levels and BMI. Male patients had a higher Framingham 10‐year CVD risk score. Table 1 presents the detailed baseline characteristics of hypertensive patients included in the study population.

**TABLE 1:** Baseline characteristics and crude end points of the study participants

	Male	Female	p‐Value
*N*	6016	3307	
Fasting HDL cholesterol, mg/dL	49.26 ± 12.40	59.44 ± 15.61	<.001
HDL quartiles			
1	35.77 ± 3.77	41.97 ± 4.30	<.001
2	43.50 ± 1.70	51.90 ± 2.54	<.001
3	50.63 ± 2.53	61.64 ± 3.10	<.001
4	65.89 ± 10.17	79.94 ± 11.97	<.001
Treatment			
Intensive, *n* (%)	2992 (49.73%)	1670 (50.50%)	.480
BMI	29.72 ± 5.27	30.09 ± 6.57	<.001
Age, y			
Overall	67.56 ± 9.34	68.53 ± 9.54	<.001
≥75y, *n* (%)	1633 (27.14%)	992 (30.00%)	.003
Race, *n* (%)			<.001
Non‐Hispanic Black	1526 (25.37%)	1259 (38.07%)	
Hispanic	528 (8.78%)	450 (13.61%)	
Other	119 (1.98%)	55 (1.66%)	
Non‐Hispanic White	3843 (63.88%)	1543 (46.66%)	
Black Race, *n* (%)	1582 (26.30%)	1348 (40.76%)	<.001
Baseline blood pressure, mmHg			
Systolic	138.80 ± 14.77	141.24 ± 16.84	<.001
Diastolic	78.40 ± 11.79	77.64 ± 12.20	<.001
Distribution of systolic blood pressure, *n* (%)			<.001
≤132 mmHg	2090 (34.74%)	1034 (31.27%)	
>132 to <145 mmHg	2043 (33.96%)	983 (29.72%)	
≥145 mmHg	1883 (31.30%)	1290 (39.01%)	
Serum creatinine, mg/dl	1.15 ± 0.33	0.95 ± 0.31	<.001
Estimated GFR, ml min^−1^ 1.73 m^−2^	72.53 ± 20.11	70.32 ± 21.37	<.001
Fasting LDL cholesterol, mg/dl	106.84 ± 33.13	122.42 ± 36.32	<.001
Fasting total cholesterol, mg/dl	181.49 ± 38.55	205.80 ± 41.15	.002
Fasting total triglycerides, mg/dl	129.09 ± 100.41	120.19 ± 68.60	<.001
Fasting glucose, mg/dl	99.78 ± 13.50	97.06 ± 13.46	<.001
Statin use, *n* (%)	2814 (47.10%)	1232 (37.48%)	<.001
Aspirin use, *n* (%)	3288 (54.81%)	1459 (44.15%)	<.001
Smoking status, *n* (%)			<.001
Never smoked	2291 (38.08%)	1820 (55.03%)	
Former smoker	2917 (48.49%)	1046 (31.63%)	
Current smoker	798 (13.26%)	440 (13.31%)	
Framingham 10‐y cardiovascular disease risk score, %	23.83 ± 10.94	13.29 ± 6.52	<.001
No. of Antihypertensive agents	1.79 ± 1.05	1.91 ± 1.02	<.001
Not using antihypertensive agents, n (%)	650 (10.80%)	230 (6.93%)	<.001
MACEs	395 (6.57%)	166 (5.02%)	.003

Abbreviations: GFR, glomerular filtration rate; HDL, high‐density lipoprotein.

*Note*: Plus–minus values are means±*SD*. To convert the values for creatinine to micromoles per liter, multiply by 88.4. To convert the values for cholesterol to millimoles per liter, multiply by 0.02586. To convert the values for triglycerides to millimoles per liter, multiply by 0.01129. To convert the values for glucose to millimoles per liter, multiply by 0.05551. Race and ethnic group were self‐reported. Black race includes Hispanic black and black as part of a multiracial identification. The body mass index is the weight in kilograms divided by the square of the height in meters.

### 3.2 Quartiles of HDL‐C levels and MACEs

The association between HDL‐C levels and MACEs in hypertensive patients is presented in Table 2. With each higher quartile of HDL‐C levels, the risk of MACEs in male patients decreased. Adjusting the model did not affect this trend. In Model 3, MACEs in the fourth quartile had the lowest risk (HR, 0.58; 95% confidence interval [CI], 0.410.82; *p* = .0023) as compared with those in the first quartile. HDL‐C levels as a continuous variable did not change this trend. Female patients in the fourth quartile of HDL‐C levels had an HR of 1.09 (95% CI, 0.62–1.93; *p* = .7678). HDL‐C levels were not associated with the risk of MACEs in female patients.

**TABLE 2:** Quartiles of HDL and MACEs

HDL quartile	Hazard ratio (95% CI) *p*‐value		
	Model 1	Model 2	Model 3
Male			
1	Ref	Ref	Ref
2	0.90 (0.69, 1.17) 0.4138	0.86 (0.66, 1.12) 0.2573	0.83 (0.63, 1.11) 0.2121
3	0.72 (0.55, 0.94) 0.0168	0.65 (0.49, 0.85) 0.0019	0.64 (0.48, 0.87) 0.0043
4	0.67 (0.50, 0.89) 0.0056	0.57 (0.43, 0.76) 0.0001	0.58 (0.41, 0.82) 0.0023
HDL quartile as a continuous variable	0.87 (0.79, 0.95) 0.0016	0.82 (0.75, 0.90) <0.0001	0.82 (0.74, 0.92) 0.0007
Female			
1	Ref	Ref	Ref
2	1.11 (0.71, 1.74) 0.6445	0.98 (0.63, 1.53) 0.9271	1.07 (0.66, 1.73) 0.7810
3	1.15 (0.73, 1.80) 0.5469	0.93 (0.59, 1.47) 0.7667	1.01 (0.61, 1.70) 0.9552
4	1.15 (0.73, 1.80) 0.5469	0.93 (0.59, 1.46) 0.7500	1.09 (0.62, 1.93) 0.7678
HDL quartile as a continuous variable	1.05 (0.92, 1.21) 0.4455	0.97 (0.85, 1.12) 0.7140	1.02 (0.85, 1.22) 0.8411

*Note*: Model 1, unadjusted; Model 2, adjusted for age, treatment arm and ethnicity; Model 3, full adjusted model, adjusted for age, treatment arm, ethnicity, baseline systolic and diastolic blood pressure, baseline body mass index, smoking status, chronic kidney disease (CKD) subgroup, cardiovascular disease (CVD) subgroup, baseline total cholesterol, baseline triglycerides, baseline urine albumin/creatinine ratio, No. of antihypertensive agents, aspirin used, and statin used.

Abbreviation: HDL‐C, high‐density lipoprotein cholesterol; MACEs, major adverse cardiovascular events; Ref, reference.

### 3.3 HDL‐C levels as a continuous variable and MACEs

As shown in Table 3, when we used HDL‐C levels as a continuous covariate, each 10 mg/dl increase in HDL‐C levels decreased the risk of MACEs in men (HR, 0.78; 95% CI, 0.70–0.88; *p* < .0001). However, HDL‐C levels were not associated with MACEs among female hypertensive patients (HR, 1.02; 95% CI, 0.89–1.16; *p* = .7869).

**TABLE 3:** Results of two‐piecewise linear‐regression model

	Male	Female	Total
One linear‐regression model	0.78 (0.70, 0.88) *p* < .0001	1.02 (0.89, 1.16) *p* = .7869	0.87 (0.80, 0.95) *p* = .0016
Inflection point (K)	44	47	44
<K Effect size β (95%CI)	0.64 (0.49, 0.86) *p* = .0024	1.18 (0.62, 2.23) *p* = .6200	0.70 (0.57, 0.87) *p* = .0011
>K Effect size β (95%CI)	0.83 (0.72, 0.95) *p* = .0076	1.01 (0.88, 1.16) *p* = .9100	0.92 (0.84, 1.01) *p* = .0889
Log likelihood ratio test	0.146	0.648	0.033

*Note*: The HR per 10 mg/dl increase in HDL‐C levels for MACEs.

**FIGURE 1:**
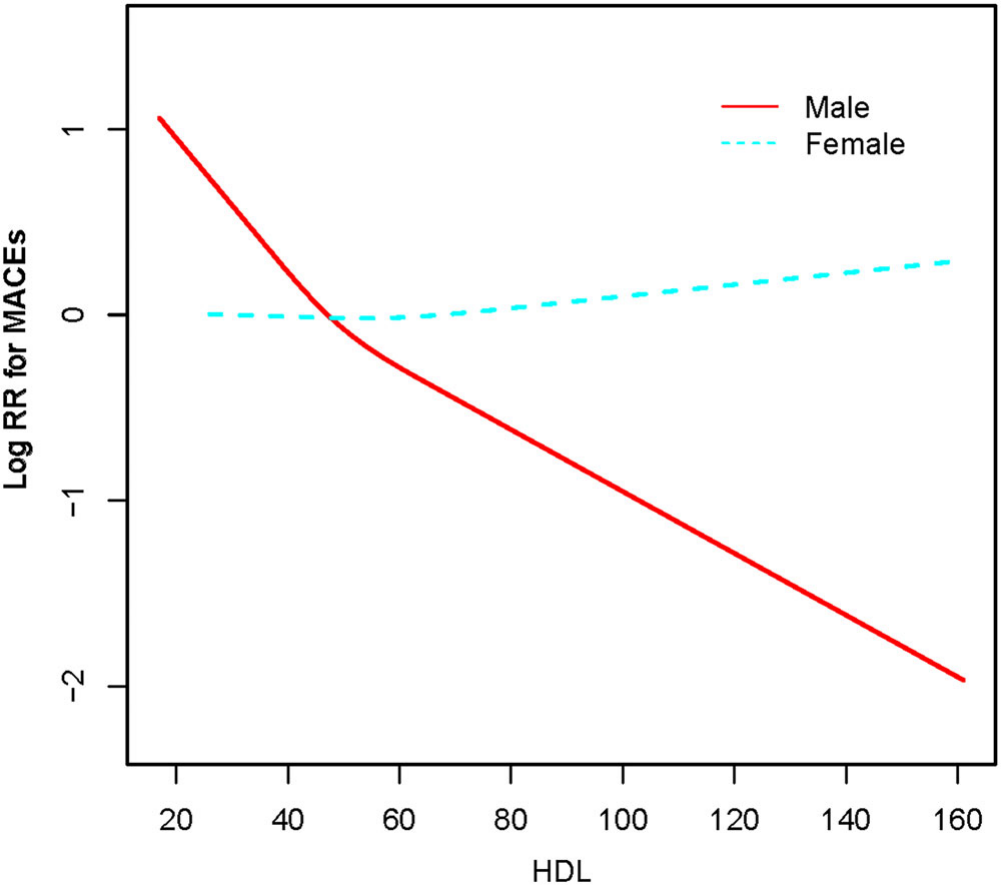
Smooth spline curves of HDL‐C levels for the estimation of risk of MACEs after adjusting multivariate rates. HDL‐C, high‐density lipoprotein cholesterol; MACEs, major adverse cardiovascular events

Restricted cubic splines were used to flexibly model and visualize the relationship between HDL‐C levels and MACEs in both male and female patients. In male patients, elevated HDL‐C levels significantly reduced the risk of MACEs. However, the risk of MACEs in women did not change with BMI (Figure 1).

Next, we used the two‐stage linear regression model to calculate the threshold effect. Table 3 shows the results of the two‐stage linear regression model. The inflection point was 44 mg/dl in all participants; on the left inflection point, the effect size, 95% CI, and *p* value were .70, .57–.87, and .0011, respectively (per 10 mg/dl increase in HDL‐C levels for MACEs). In female participants, HDL‐C levels were not associated with MACEs in the two‐stage linear regression model or one‐line linear regression model. In male participants, when the HDL‐C levels were lower than 44 mg/dl, the risk of MACEs significantly decreased per 10 mg/dl increase in HDL‐C levels (HR, 0.64; 95% CI, 0.49–0.86; *p* = .0024). When the HDL‐C levels were greater than 44 mg/dl, the risk of MACEs also decreased per 10 mg/dl increase in HDL‐C levels (HR, 0.83; 95% CI, 0.72–0.95; *p* = .0076), although the downward trend was slower.

### 3.4 LDL‐C levels and MACEs

The association between LDL‐C levels and MACEs in hypertensive patients is shown in Table S1. With each higher quartile of LDL‐C levels, the risk of MACEs in male patients decreased. Adjusting the model did not affect this trend. In Model 3, MACEs in the fourth quartile had the highest risk (HR, 1.24; 95% CI, 1.01–1.62; *p* = .0371) as compared with those in the first quartile. Quartiles of LDL‐C levels as a continuous variable did not change this trend. This trend was not observed in female patients. Female patients in the third quartile had the lowest risk of MACEs (HR, 0.48; 95% CI, 0.26–0.88; *p* = .0175, Model 3).

As shown in Table S2, when we used LDL‐C levels as a continuous covariate, each 10 mg/dl increase in LDL‐C levels increased the risk of MACEs in men (HR, 1.25; 95% CI, 1.11–1.40; *p* < .0002) and all participants (HR, 1.12; 95% CI, 1.02–1.22; *p* < .0124). Nevertheless, LDL‐C levels were not associated with MACEs in female hypertensive patients (HR, 0.96; 95% CI, 0.84–1.09; *p* = .5265). In female participants, when the LDL‐C levels were lower than 110 mg/dl, the risk of MACEs significantly decreased per 10 mg/dl increase in LDL‐C levels (HR, 0.82; 95% CI, 0.70–0.96; *p* = .0164) (Figure S1). When the LDL‐C levels were greater than 110 mg/dl, the risk of MACEs did not show statistical correlation with LDL‐C levels.

### 3.5 Interaction and sensitivity analyses

**FIGURE 2:**
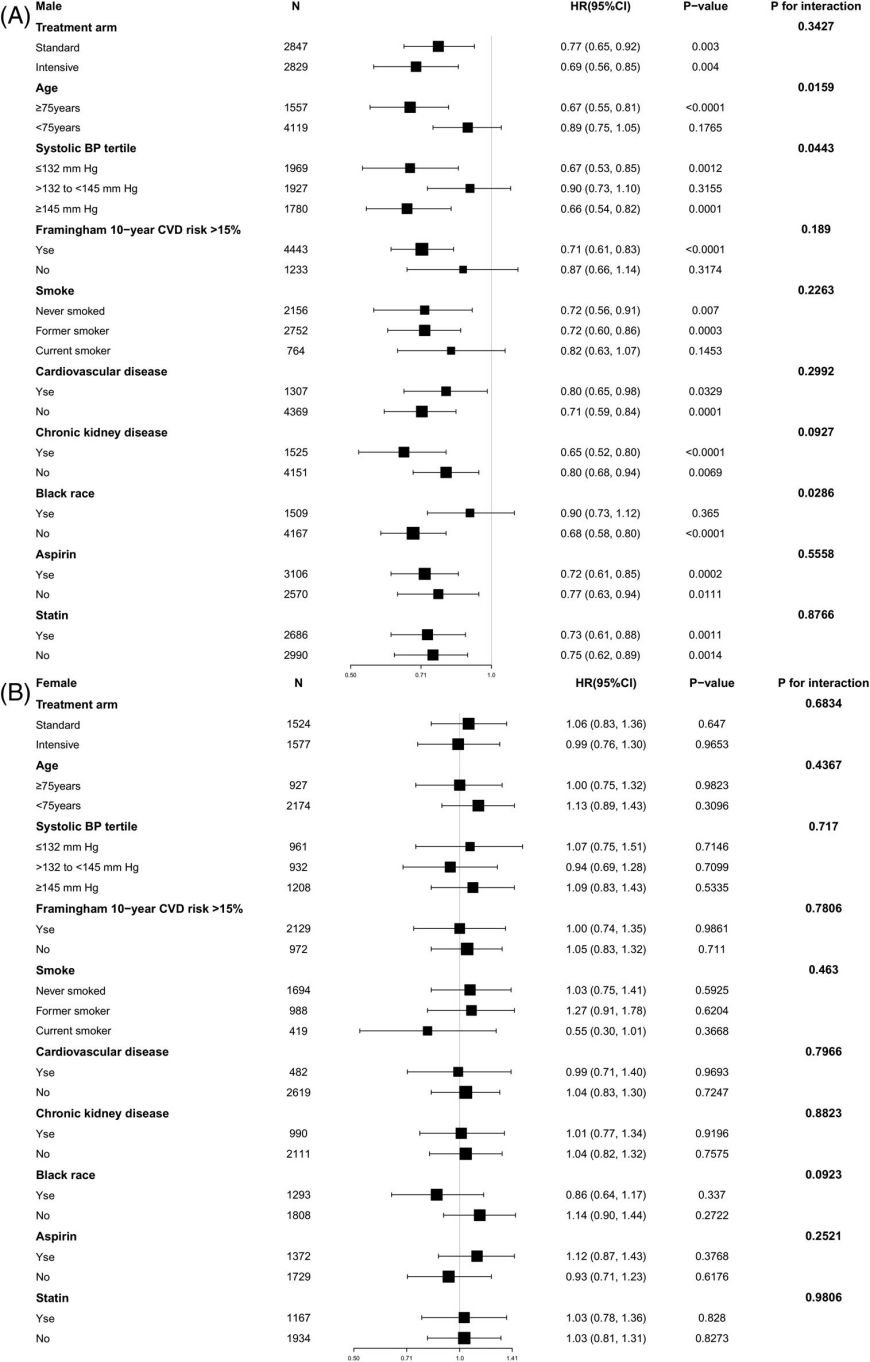
The HR per SD increase in HDL‐C for MACEs. (A) Data for male hypertension patients is shown. (B) Data for female hypertension patients are shown. Each stratification was adjusted for all factors in Model 3, except for the stratification factor itself. HDL‐C, high‐density lipoprotein cholesterol; HR, hazard ratio; MACEs, major adverse cardiovascular events

The results of the interaction and stratified analyses are presented in Figure 2. Age and SBP tertile played an interactive role in the association between HDL‐C levels and MACEs among male patients (*p* = .0159 and *p* = .0143, respectively). In men aged <75 years, elevated HDL‐C levels did not reduce the risk of MACEs (HR per SD increase in HDL‐C levels: 0.89; 95% CI, 0.75–1.05; *p* = .1765), whereas in men aged >75 years, the risk of MACEs significantly decreased with elevated HDL‐C levels (HR per SD increase in HDL‐C levels: 0.67; 95% CI, 0.55–0.81; *p* < .0001). HDL‐C levels were not associated with the risk of MACEs in patients with SBP between 132 and 145 mmHg (HR per SD increase in HDL‐C levels: 0.90; 95% CI, 0.73–1.10; *p* = .3155). We did not find interactions between HDL‐C levels and other confounders in male patients. There was no factor that played an interactive role in the association between HDL‐C levels and MACEs in female patients.

## 4 DISCUSSION

In this post‐hoc analysis involving 9323 hypertensive patients from the SPRINT, we found a significant sex difference in the relationship between HDL‐C levels and adverse cardiovascular events. In male patients, high HDL‐C levels predicted a lower incidence of cardiovascular outcomes, whereas in female patients, HDL‐C levels were not associated with the incidence of adverse cardiovascular events.

Several previous studies have shown that HDL‐C levels are inversely associated with both CVD and mortality across a wide range of concentrations.^16^, ^17^ However, some recent studies have cast doubt on the cardioprotective effect of HDL; they reported that the use of drugs to increase HDL levels did not provide the expected cardiovascular protection.^18^, ^19^ A meta‐analysis of 299 310 participants at risk for cardiovascular events showed that increasing the circulating HDL‐C levels did not reduce the risk of coronary heart disease events, coronary heart disease death, or all‐cause death.^20^ There were similar inconsistencies among reports using experimental animal models: transgenic‐induced elevation of HDL‐C levels increased atherosclerosis, and gene transfer resulting in significantly lower HDL‐C levels led to excessive HDL receptor expression and reduced atherosclerosis. This highlights the complexity of the relationship between HDL‐C levels and CVD. HDL‐C levels may not be able to fully reflect the real cardioprotective effect of HDL.^21^

Madsen et al. revealed that the relationship between HDL‐C levels and all‐cause mortality was U‐shaped and reported that men and women with very high HDL‐C levels in the general population had higher all‐cause mortality. The relationship was most pronounced in men and those with cardiovascular mortality.^22^ However, this finding conflicts with the results of our study. This might be due to differences in the population of the participants. Madsen et al. studied a group from a general population. Participants in the SPRINT were all hypertensive patients with multiple risk factors and were, on average, significantly older.

It is well known that HDL loses its cardiovascular protective function under certain conditions and may promote the development of atherosclerosis, especially in postmenopausal women.^21^, ^23^, ^24^, ^25^ This might be related to changes in hormone levels that occur after menopause. The transition to menopause in women is affected by several adverse physiological changes, including changes in sex hormones, body fat deposition, and lipid distribution. Over time, the accumulation of these changes may trigger a chronic inflammatory state that may hinder the cardioprotective ability of HDL.^16^, ^26^

Menopausal hormone changes could cause the accumulation of risk factors for CVD. The reduction of estradiol, an effective antioxidant, can increase lipid peroxidation and reactive oxygen species formation in women during the menopausal transition. This in turn might affect HDL protein composition, thereby depleting anti‐inflammatory and/or antioxidant proteins and enriching pro‐inflammatory proteins. In vitro biochemical analysis of lipoprotein in a small subset of premenopausal and postmenopausal women (30 per group) showed that postmenopausal women displayed impairment in their ability to limit LDL oxidation, independent of HDL. These results suggest that HDL particles may lose some of their anti‐atherosclerotic properties during the menopausal transition.^27^, ^28^, ^29^

El Khoudary et al. reported that menopause may have differing effects on different HDL particles. They found that menopause does not affect the cardioprotective effect of small HDL particles, whereas large HDL particles are affected by menopause. They suggest that high HDL‐C levels in older women may be a marker of HDL dysfunction. This may be one of the reasons why HDL‐C loses its cardioprotective effect in older women.^12^

As for LDL‐C, through meta‐analysis, the CTT study has shown since 2010 that a decrease in the absolute value of LDL‐C will lead to a significant decrease in atherosclerotic CVD (ASCVD) endpoint events. Subsequent studies, including the IMPROVE‐IT, FOURIER, and ODYSSEY OUTCOMES trials, have confirmed that a decrease in the absolute value of LDL‐C will result in a decrease in ASCVD endpoint events.^30^, ^31^, ^32^ In a study involving 27 533 women with an average follow‐up period of 17.2 years, Mora et al. revealed that relying on LDL‐C alone might overestimate or underestimate the risk in a subgroup in which LDL‐C was inconsistent with another LDL‐related measurement method (up to a quarter of the total population). Therefore, in our study, no statistically significant correlation between LDL‐C levels and the risk of MACEs was identified among female patients.^33^ This may be attributable to the short follow‐up period or to the fact that the LDL‐C level itself is not a good predictor of MACEs.

This post‐hoc analysis has some limitations. First, this was a retrospective study, and the original study was not designed to examine the relationship between HDL‐C levels and adverse cardiovascular events. Second, we did not obtain data on HDL function in patients. Finally, we analyzed the patients' baseline HDL‐C levels; although we adjusted the LDL‐C levels in the analysis, we were still unable to control for all variables that might have influenced the results.

## 5 CONCLUSION

In elderly hypertensive patients, HDL‐C levels were associated with MACEs in male but not female patients. Higher HDL‐C levels were associated with a lower MACE incidence in men. In contrast, HDL‐C levels were not associated with MACE incidence in women.

## CONFLICT OF INTEREST

The authors declare that they have no competing interests.

## AUTHOR CONTRIBUTIONS

Xinqun Hu and Xiaopu Wang designed the study and provided methodological expertise. Xiaopu Wang drafted the manuscript. Xiaopu Wang, Junyu Pei, Keyang Zheng, and Xinqun Hu drafted the tables and figures and performed statistical analysis. Junyu Pei revised the manuscript. All authors have read and approved the final manuscript.

## Supporting information


**Figure S1** Smooth spline curves of LDL‐C levels for the estimation of risk of MACEs after adjusting multivariate rates. MACEs, major adverse cardiovascular events.

**Table S1** Quartiles of LDL and MACEs

**Table S2** Results of two‐piecewise linear‐regression model.

## Data Availability

The data that support the findings of this study are available from Biologic Specimen and Data Repository Information Coordinating Center (BioLINCC). Restrictions apply to the availability of these data, which were used under license for this study. Data are available [https://biolincc.nhlbi.nih.gov/studies/sprint/] with the permission of BioLINCC.
