# Supplementary figures and images for: High‐density lipoprotein cholesterol levels are associated with major adverse cardiovascular events in male but not female patients with hypertension
Source: Clin Cardiol. 2021 Mar 30;44(5):723–30. doi: 10.1002/clc.23606 (PMC8119833; doi:10.1002/clc.23606)

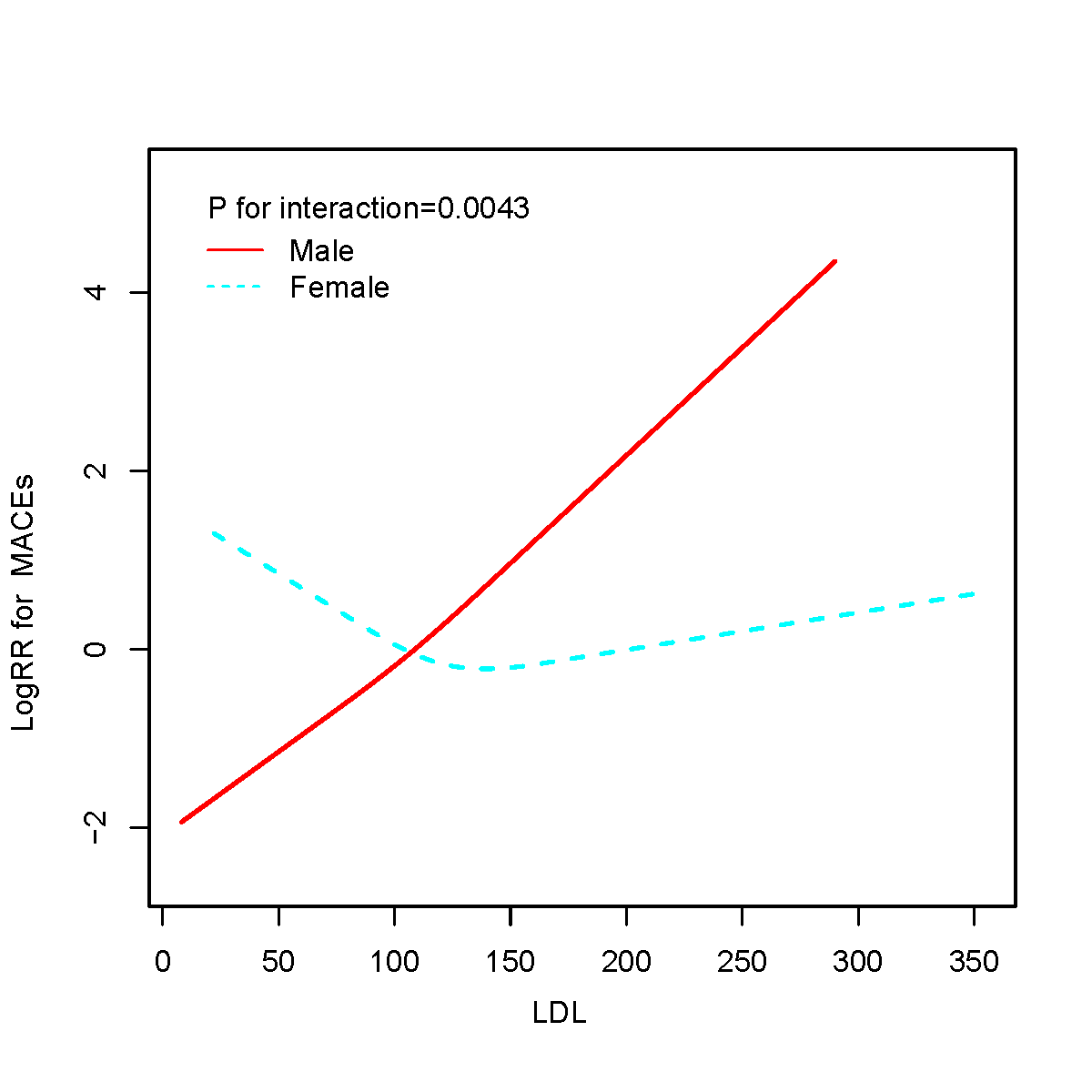

Supplement: Supplementary file 1 — Figure S1 Smooth spline curves of LDL‐C levels for the estimation of risk of MACEs after adjusting multivariate rates. MACEs, major adverse cardiovascular events. [file CLC-44-723-s001.tiff]
