# Supplementary material for: High‐density lipoprotein cholesterol levels are associated with major adverse cardiovascular events in male but not female patients with hypertension
Source: Clin Cardiol. 2021 Mar 30;44(5):723–30. doi: 10.1002/clc.23606 (PMC8119833; doi:10.1002/clc.23606)
Supplement: Supplementary file 2 — Table S1 Quartiles of LDL and MACEs [file CLC-44-723-s002.docx]

Table S1 Quartiles of LDL and MACEs

| LDL quartile | Hazard ratio (95% CI) *P*-Value | | |
| --- | --- | --- | --- |
|  | Model 1 | Model 2 | Model 3 |
| Male |  |  |  |
| 1 | Ref | Ref | Ref |
| 2 | 0.81 (0.62, 1.05) 0.1088 | 0.89 (0.68, 1.16) 0.3824 | 1.19 (0.85, 1.65) 0.3095 |
| 3 | 0.73 (0.55, 0.97) 0.0300 | 0.90 (0.67, 1.20) 0.4753 | 1.34 (0.86, 2.10) 0.1980 |
| 4 | 0.98 (0.74, 1.28) 0.8579 | 1.27 (0.96, 1.67) 0.0975 | 2.11 (1.13, 3.95) 0.0188 |
| LDL quartile as a continuous variable | 0.97 (0.89, 1.06) 0.5438 | 1.07 (0.97, 1.17) 0.1818 | 1.24 (1.01, 1.52) 0.0371 |
| Female |  |  |  |
| 1 | Ref | Ref | Ref |
| 2 | 0.58 (0.38, 0.91) 0.0164 | 0.60 (0.39, 0.94) 0.0252 | 0.68 (0.42, 1.12) 0.1307 |
| 3 | 0.38 (0.24, 0.60) <0.0001 | 0.42 (0.26, 0.67) 0.0003 | 0.48 (0.26, 0.88) 0.0175 |
| 4 | 0.58 (0.39, 0.86) 0.0071 | 0.72 (0.48, 1.07) 0.1049 | 0.75 (0.34, 1.66) 0.4729 |
| LDL quartile as a continuous variable | 0.58 (0.39, 0.86) 0.0071 | 0.89 (0.77, 1.03) 0.1082 | 0.84 (0.65, 1.10) 0.2104 |

Model 1, unadjusted; model 2, adjusted for age, treatment arm and ethnicity; model 3, full adjusted model, adjusted for age, treatment arm, ethnicity, baseline systolic and diastolic blood pressure, baseline body mass index, smoking status, chronic kidney disease (CKD) subgroup, cardiovascular disease (CVD) subgroup, baseline total cholesterol, baseline triglycerides, baseline urine albumin/creatinine ratio, No. of antihypertensive agents, aspirin used and statin used. Ref, reference.
