# Supplementary material for: High‐density lipoprotein cholesterol levels are associated with major adverse cardiovascular events in male but not female patients with hypertension
Source: Clin Cardiol. 2021 Mar 30;44(5):723–30. doi: 10.1002/clc.23606 (PMC8119833; doi:10.1002/clc.23606)
Supplement: Supplementary file 3 — Table S2 Results of two‐piecewise linear‐regression model. [file CLC-44-723-s003.docx]

**Table S2. Results of two-piecewise linear-regression model.**

|  | Male | Female | Total |
| --- | --- | --- | --- |
| One linear-regression model | 1.25 (1.11, 1.40) *P*<0.0002 | 0.96 (0.84, 1.09) *P*=0.5265 | 1.12 (1.02, 1.22) *P*=0.0124 |
| Inflection point (K) | 110 | 110 | 11 |
| <K Effect size β (95%CI) | 1.21 (1.06, 1.38) *P*=0.0051 | 0.82 (0.70, 0.96) *P*=0.0164 | 1.06 (0.96, 1.17) *P*=0.2834 |
| >K Effect size β (95%CI) | 1.28 (1.13, 1.45) *P*=0.0001 | 1.03 (0.90, 1.17) *P*=0.7170 | 1.15 (1.05, 1.26) *P*=0.0022 |
| Log likelihood ratio test | 0.268 | 0.003 | 0.035 |

The HR per 10 mg/dL increase in LDL-C levels for MACEs.
